# Supplementary material for: Analysis of Dibenzyltoluene Mixtures: From Fast Analysis to In-Depth Characterization of the Compounds
Source: Molecules. 2023 Apr 27;28(9):3751. doi: 10.3390/molecules28093751 (PMC10180323; doi:10.3390/molecules28093751)
Supplement: Supplementary file 1 [file molecules-28-03751-s001.zip › molecules-2349297-supplementary.pdf]

Supporting information

Analysis of dibenzyltoluene mixtures, from fast analysis to deep characterization of the compounds

Xiaolong Ji[1,2], Essyllt Louarn[1,3*], Fabienne Fache[4], Laurent Vanoye[5], Anne Bonhommé[1], Isabelle Pitault[2] and Valérie Meille[1,*]

[1] Univ Lyon, Université Claude Bernard Lyon 1, CNRS, IRCELYON, F-69626, Villeurbanne, France; valerie.meille@ircelyon.univ-lyon1.fr

[2] Univ Lyon, Université Claude Bernard Lyon 1, CNRS, LAGEPP, F-69100, Villeurbanne, France; isabelle.pitault@univ-lyon1.fr

[3] Université Paris Saclay, CNRS, ICP, F-91405, Orsay, France; essyllt.louarn@universite-paris-saclay.fr

[4] Univ Lyon, Université Claude Bernard Lyon 1, CNRS, ICBMS, F-69100, Villeurbanne, France

[5] Université de Lyon, Institut de Chimie de Lyon, Laboratory of Catalysis, Polymerization, Processes & Materials, CP2M UMR 5128 CNRS-UCB Lyon 1-CPE Lyon, CPE Lyon 43 Bd du 11 Novembre 1918, F-69616, Villeurbanne, France

[*] Correspondence: essyllt.louarn@universite-paris-saclay.fr (E.L.), valerie.meille@ircelyon.univ-lyon1.fr (V.M.)

SI-1 Relative response factors (RRFs) of Hx-DBT families

Using octadecane (C18) as standard, the relative response factors of H0-DBT was obtained using the slope of C18/H0-DBT GC integration area ratio (A(H0-DBT)/A(C18)) over mass ratio (m(H0-DBT)/m(C18)) as shown in figure SI-1. Different samples with different H0-DBT/C18 mass ratios were prepared. Pure H0 compound was used for its calibration. Each sample was injected 3 times into the GC consecutively to determine the error of the GC instrument and method, which was shown to be very small. The RRFs of H6, H12 and H18-DBT were supposed to be close to that of H0-DBT as a first approach.

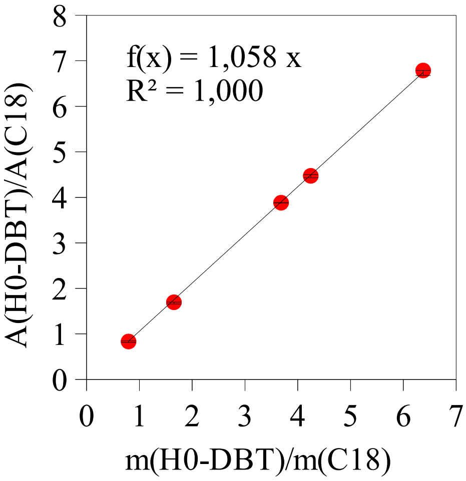

Figure S1. Calibration curve of H0-DBT

SI-2 GC/MS chromatogram of concentrated H0-DBT sample

A more concentrated H0-DBT sample was prepared and analyzed by the GC/MS apparatus to better show the presence of minor pics in the mixture as shown in figure SI-2.

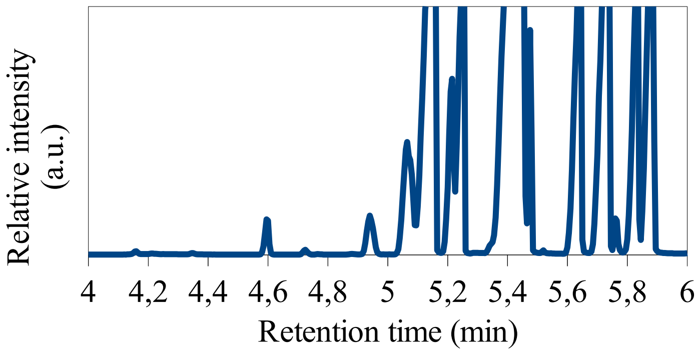

Figure S2. GC/MS chromatogram of concentrated H0-DBT

SI-3 Detailed MS table of H0-DBT isomers

Mass spectra for each peak identified on the chromatogram and those assigned in the literature (K90 [1] and M00 [2]). Our results show the number of the assigned peak, the corresponding retention time (tr in min), the height of the peak signal in the chromatogram (TIC). The first three retention times correspond to phenylditoluylmethane (PdTM). In bold, attribution confirmed by syntheses.

Peak No.	Attribution	Tr (min)	TIC	Ion mass over charge (m/z)												
				272	257	195	194	181	180	179	167	166	165	105	104	91
1	PdTM	4.610	<LOQ	71	100	10		29	21	39		29	65			
2		4.745	<LOQ	100	88					85			99			
3		4.950	34165	78	100	30		16	18	47		28	79			8
4	2,3 DBT	5.063	82042	41	2	4	16	31	17	100	4	21	28			17
5	3,2' BBT	5.083	30024	60			12	47	83	100	23	25	58			27
6	**3,4 DBT**	5.115	259092	51		4	21	50	15	100	5	27	33			17
		K90(3)		100	3			90			15	70				90
7	2,2' BBT	5.188	70612	12				19	100	85	7	13	36	4	6	14
		K90(2)		100	9			60			40		50		40	
		M00(a)		82				-	85	100						-
8	4,2' BBT	5.220	121124	31			4	21	84	100	9	15	38	6		11
		K90(4)		100	10			95			37		60	15		50
		M00(b)		42	10			-	100	86						-
9	**3,5 DBT**	5.293	<LOQ	62				100								
		M00(c)		58				100								15
10	2,3' BBT	5.343	40338	94	10			14	21	23	80	26	56	33	100	46
11	**2,4 DBT**	5.370	621877	51	4	1	4	100	3	15	4	29	30			31
		K90(1)		40				35		100			48			22
		M00(e)		62				10	-	-						17
12	3,4' BBT	5.438	52165	73	7		5	100	8	23	44	35	45	19		47
13	**2,5 DBT**	5.608	138433	56	4		4	100	5	30	5	32	33			42
		K90(5)		100	3			97				40	45			50
14	2,6 DBT	5.695	228073	50	1	1	2	100	3	14	5	32	31			31
		K90(6)		95				100			25		30			10
15	4,3' DBT	5.750	33111	74				98			100	56	51			35
		K90(7)		100	10			85			50		40	15		10
16	2,4' BBT	5.803	118206	100	15			98	8	20	89	43	65	24	95	54
		K90(8)		100	11			60			50		40		40	

		M00(f)		62				10	-	-						17
17	4,4' BBT	5.843	157482	75	13		2	100	6	14	61	33	46	16	5	34
		K90(9)		50				50	60	70			70	13		100
		M00(d)		70				100	-	-						18

[1] Katritzky, A.R.; Balasubramanian, M.; Siskin, M. Aqueous high-temperature chemistry of carbo- and heterocycles. 2. Monosubstituted benzenes: benzyl alcohol, benzaldehyde and benzoic acid. *Energy & Fuels* 1990, *4*, 499–505. doi:10.1021/ef00023a016

[2] Miranda, R.; Delgado, F.; Velasco, L.; Pérez, J.; Salmón, M. Mass spectrometric detection and identification of ortho, para-benzyltoluenes and oligotoluenes. *Rapid Communications in Mass Spectrometry* **2000**, *14*, 188–193. doi:10.1002/(SICI)1097-0231(20000215)14:3<188::AID-RCM858>3.0.CO;2-O

SI-4 NMR spectra for synthesized molecules

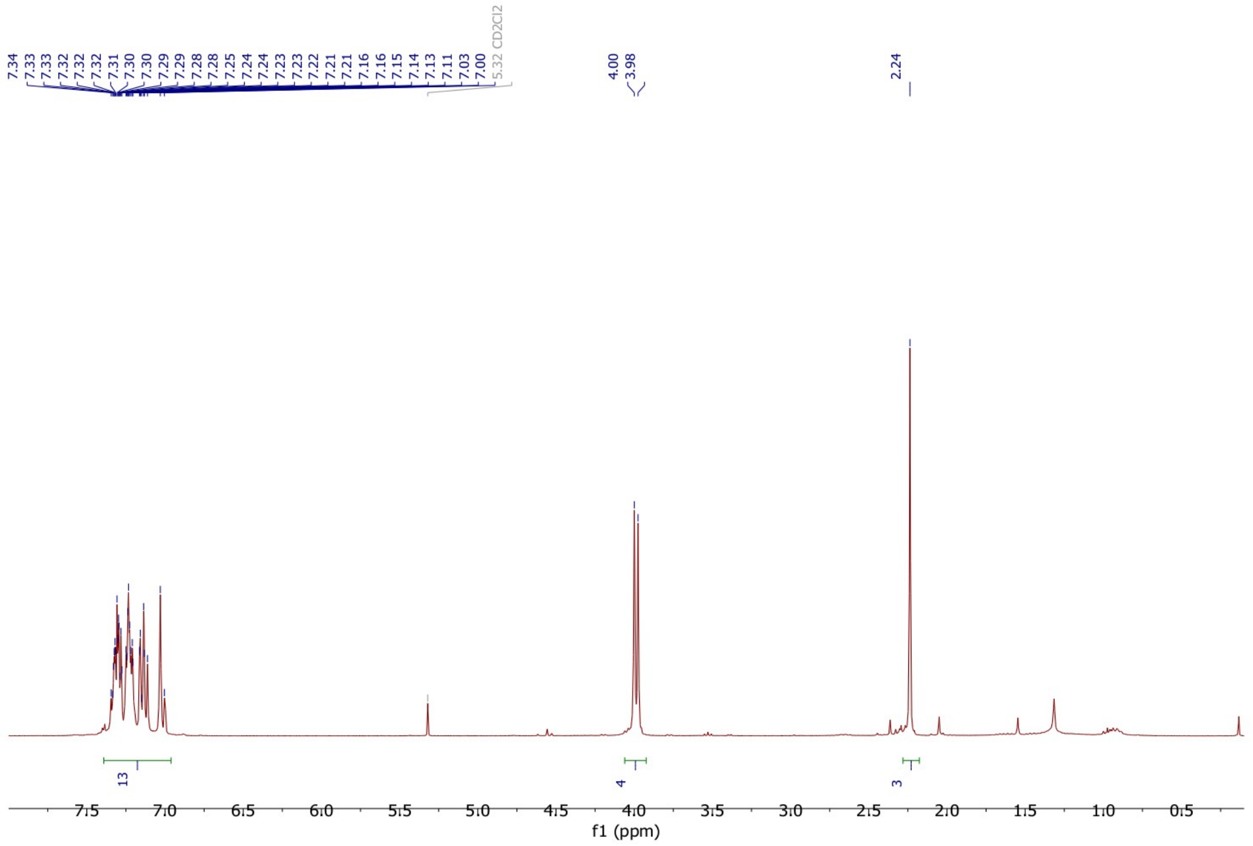

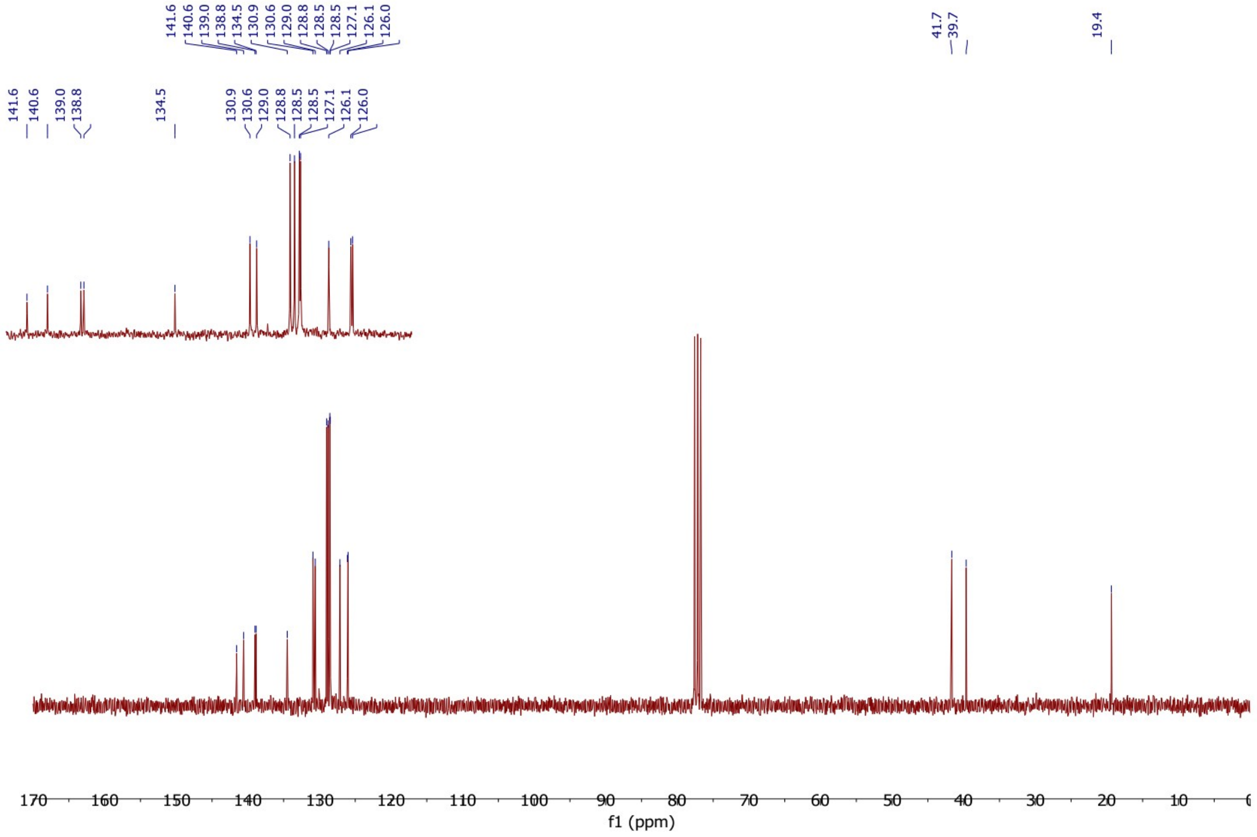

Figure S3. Top) ^{1}H and bottom) ^{13}C NMR spectra of synthesized 2,4-DBT

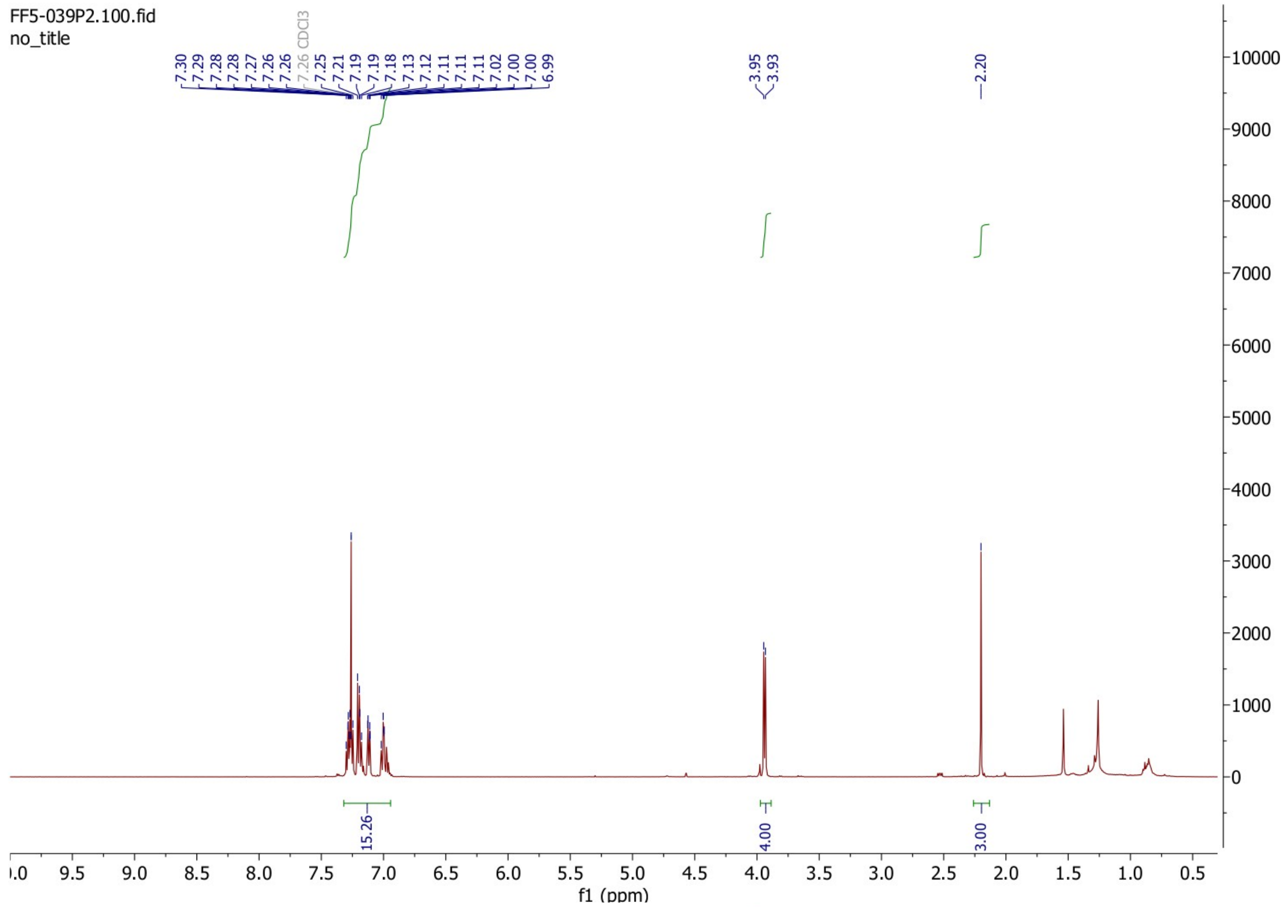

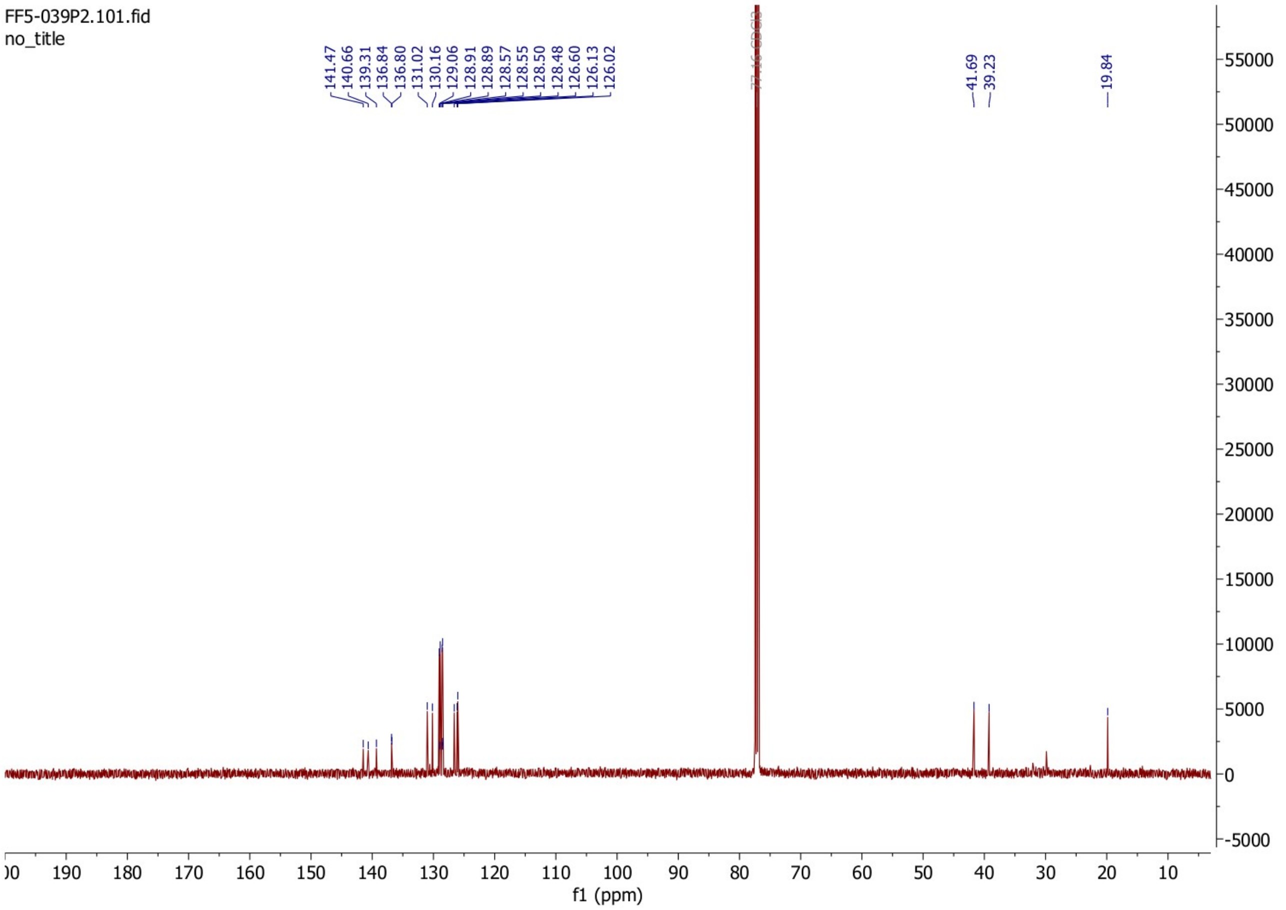

Figure S4. Top) ^{1}H and bottom) ^{13}C NMR spectra of synthesized 2,5-DBT

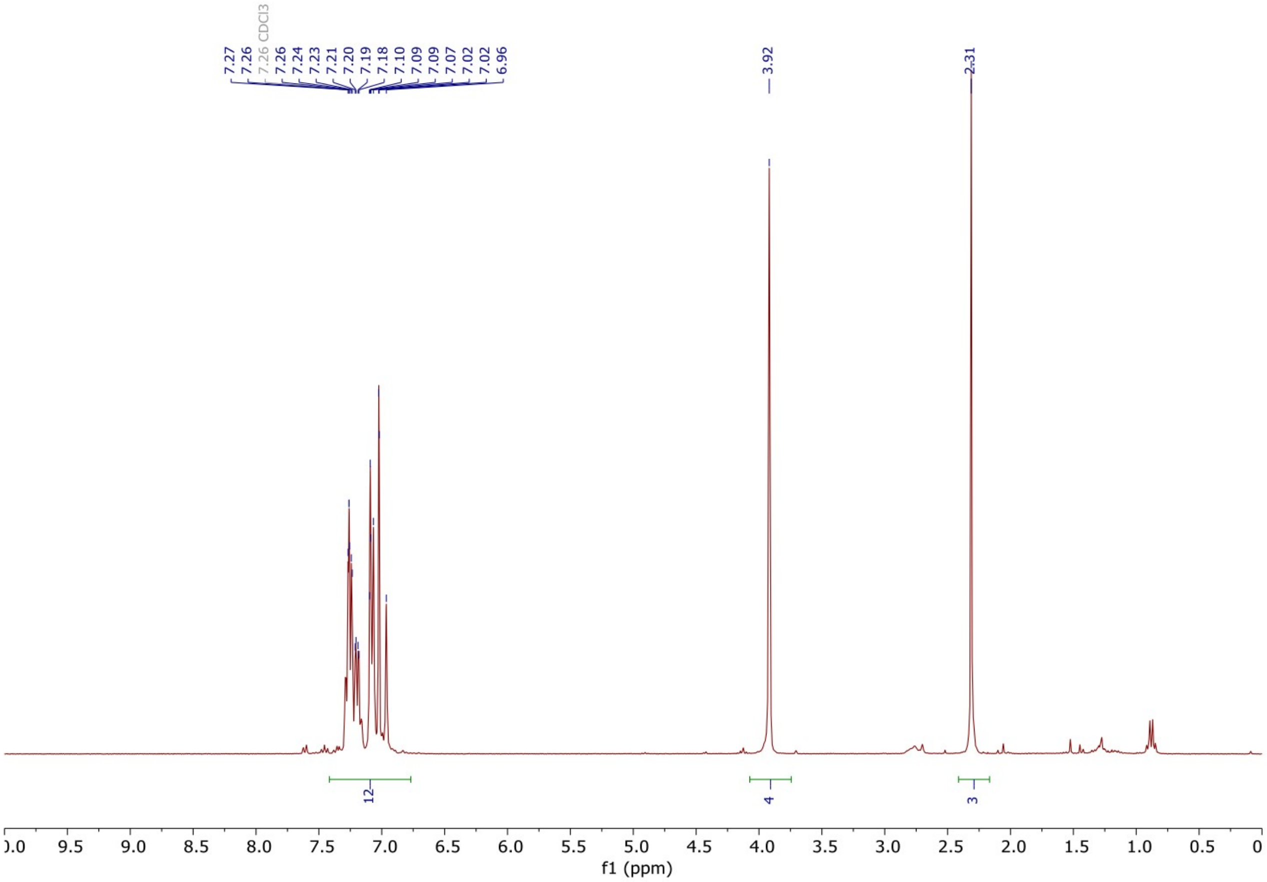

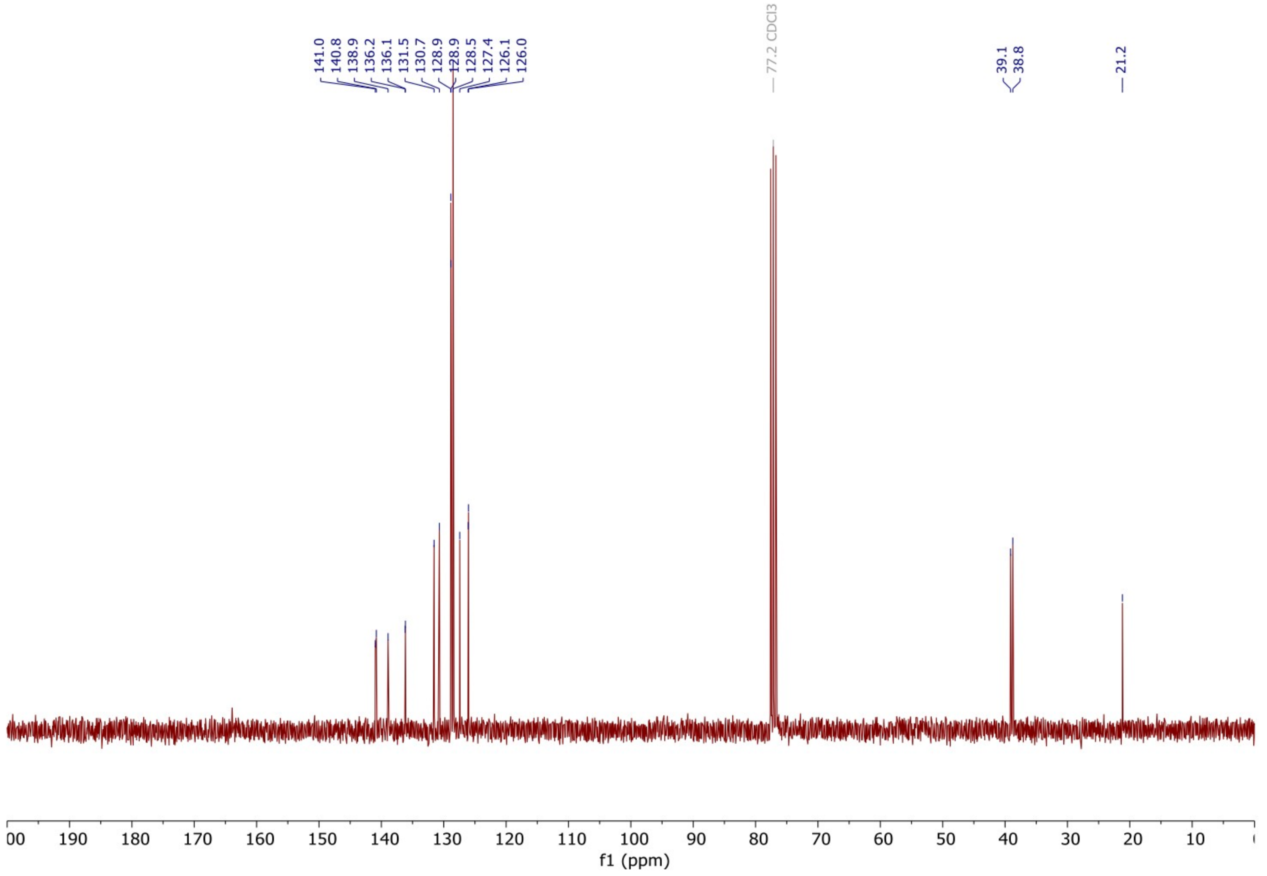

Figure S5. Top) ^{1}H and bottom) ^{13}C NMR spectra of synthesized 3,4-DBT

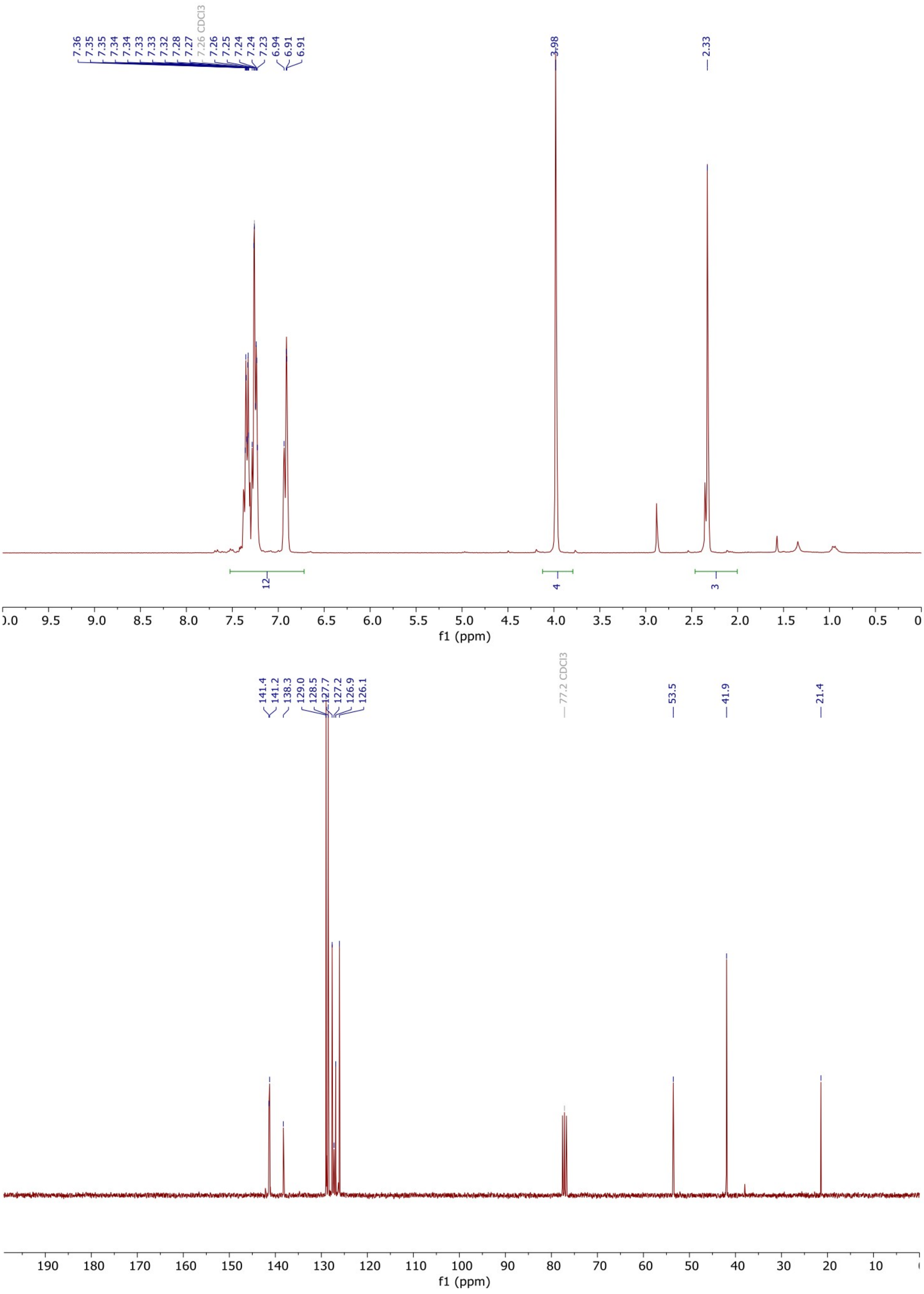

Figure S6. Top) ^{1}H and bottom) ^{13}C NMR spectra of synthesized 3,5-DBT
